# *Leptospira* Is an Environmental Bacterium That Grows in Waterlogged Soil

**DOI:** 10.1128/spectrum.02157-21

**Published:** 2022-03-15

**Authors:** Yasutake Yanagihara, Sharon Y. A. M. Villanueva, Naoki Nomura, Marumi Ohno, Toshiki Sekiya, Chimuka Handabile, Masashi Shingai, Hideaki Higashi, Shin-ichi Yoshida, Toshiyuki Masuzawa, Nina G. Gloriani, Mitsumasa Saito, Hiroshi Kida

**Affiliations:** a International Institute for Zoonosis Control, Hokkaido University, Sapporo, Japan; b University of Shizuoka, Shizuoka, Japan; c Department of Medical Microbiology, College of Public Health, University of the Philippines–Manila, Manila, Philippines; d International Collaboration Unit, International Institute for Zoonosis Control, Hokkaido University, Sapporo, Japan; e Department of Bacteriology, Graduate School of Medical Sciences, Kyushu University, Fukuoka, Japan; f Laboratory of Microbiology and Immunology, Faculty of Pharmaceutical Sciences, Chiba Institute of Science, Chiba, Japan; g Institute of Pathology, St Luke’s Medical Center, Quezon City, Philippines; h Department of Microbiology, School of Medicine, University of Occupational and Environmental Health, Kitakyushu, Japan; i Collaborating Research Center for the Control of Infectious Diseases, Nagasaki University, Nagasaki, Japan; University of Texas at San Antonio

**Keywords:** *Leptospira*, leptospirosis, waterlogged soil, survival in soil

## Abstract

Leptospirosis is a zoonotic disease caused by infection with pathogenic leptospires. Consistent with recent studies by other groups, leptospires were isolated from 89 out of 110 (80.9%) soil or water samples from varied locations in the Philippines in our surveillance study, indicating that leptospires might have a life cycle that does not involve animal hosts. However, despite previous work, it has not been confirmed whether leptospires multiply in the soil environment under various experimental conditions. Given the fact that the case number of leptospirosis is increased after flood, we hypothesized that waterlogged soil, which mimics the postflooding environment, could be a suitable condition for growing leptospires. To verify this hypothesis, pathogenic and saprophytic leptospires were seeded in the bottles containing 2.5 times as much water as soil, and bacterial counts in the bottles were measured over time. Pathogenic and saprophytic leptospires were found to increase their number in waterlogged soil but not in water or soil alone. In addition, leptospires were reisolated from soil in closed tubes for as long as 379 days. These results indicate that leptospires are in a resting state in the soil and are able to proliferate with increased water content in the environment. This notion is strongly supported by observations that the case number of leptospirosis is significantly higher in rainy seasons and increased after flood. Therefore, we reached the following conclusion: environmental soil is a potential reservoir of leptospires.

**IMPORTANCE** Since research on *Leptospira* has focused on pathogenic leptospires, which are supposed to multiply only in animal hosts, the life cycle of saprophytic leptospires has long been a mystery. This study demonstrates that both pathogenic and saprophytic leptospires multiply in the waterlogged soil, which mimics the postflooding environment. The present results potentially explain why leptospirosis frequently occurs after floods. Therefore, environmental soil is a potential reservoir of leptospires and leptospirosis is considered an environment-borne as well as a zoonotic disease. This is a significant report to reveal that leptospires multiply under environmental conditions, and this finding leads us to reconsider the ecology of leptospires.

## INTRODUCTION

Leptospirosis is an important, but often ignored, global and life-threatening zoonosis caused by spirochete *Leptospira*. Pathogenic leptospires cause severe damage to humans, with more than one million cases and approximately 60,000 deaths annually worldwide as estimated in 2015 (1, 2).

Leptospires are distributed worldwide and have been isolated from various vertebrate species and environmental soil and water (3, 4). On the basis of the phylogenetic analyses of 16S or 23S rRNA sequences, *Leptospira* is classified into two major clades: pathogenic (P) and saprophytic (S). The pathogenic clade includes two subclades: P1 (formerly known as the pathogenic group) and P2 (the intermediate group). The saprophytic (S) clade also has two subclades: S1 and S2. Detection of *fla*B gene by PCR is utilized to easily determine whether the leptospires belong to pathogenic clades (5–7).

Pathogenic leptospires have a complex life cycle. The bacteria establish chronic infection in the renal tubules of host animals, such as rodents and livestock, without causing significant symptoms. The pathogen is excreted through the urine (8, 9), and the excreted pathogenic leptospires can survive for several months in soil and water in the absence of animal host (3, 10). According to previous reports, leptospires survived up to 593 days in freshwater (11) and 183 days in water-saturated soil (12). Given that contact with colonized soil or water is a risk factor for infection with leptospires (8, 9, 13, 14) and that outbreaks of leptospirosis are predominant during the rainy season in the Philippines, Sri Lanka, Reunion Island, Puerto Rico, and Thailand (15–19), environmental soil and water are thought to be a reservoir and courier of the pathogen. This notion is supported by a previous report that pathogenic leptospires found in the surrounding environment were genetically identical to those isolated from patients for several weeks following an outbreak of human leptospirosis (20). On the other hand, saprophytic leptospires, which account for more than half species of the *Leptospira* genus (21), have been known to be frequently isolated only from soil and water in the environment and not from animals, suggesting that the life cycle of saprophytic leptospires can be completed solely in the environment. However, it is still unclear whether or not pathogenic and even saprophytic leptospires multiply in soil or water (3, 10, 22).

Based on the fact that the case number of leptospirosis increases after flood (23–26), we hypothesized that waterlogged soil, which mimics the postflooding environment, could be a suitable condition for growing leptospires. Therefore, we conducted the growth experiments of pathogenic and saprophytic leptospires using waterlogged soil containing 2.5 times as much water as soil, and bacterial counts in the bottles were measured over time. Pathogenic and saprophytic leptospires were found to increase their number in waterlogged soil but not in water or soil alone. The results of this study provide new insights into the ecology of *Leptospira* species.

## RESULTS

### Leptospires survive for an extended time in the environment.

We collected 110 samples (106 soil and 4 water) in the Philippines and 36 samples (31 soil and 5 water) in Japan (Table 1 and Table S1). The presence of leptospires in the soil and water samples was determined by the isolation of bacteria with characteristic helical morphology and PCR amplification of *Leptospira*-specific *rrl* or sequencing of the *rrs* genes. Additionally, the *fla*B gene associated with the pathogenic (P) clade of *Leptospira* was detected by PCR. The presence of *Leptospira* was confirmed in 86 of 110 samples (78%; 83 of 106 soil samples and 3 of 4 water samples) in the Philippines and 15 of 36 samples (42%; 13 of 31 soil samples and 2 of 5 water samples) in Japan. Of 101 culture-positive samples, 21 (21%) were positive for *fla*B PCR. Environmental samples in the Philippines showed a higher positivity rate of leptospires than those in Japan, which was consistent with previous reports (Table 1) (1, 27). Interestingly, in the collection of leptospires from the environment, leptospires were detected even in the sample collected from harsh environments, such as forest soil at an altitude of more than 1,200 m in Mt. Yatsugatake and in soil (−2°C) under a 40-cm-thick layer of snow in Sapporo, where chances of contamination by leptospires from infected animals are minimal. These results suggest that leptospires survive for an extended time in the soil, as reported in water (11, 12).

**TABLE 1:** Detection of leptospires from the environment

Place	No. of leptospires in:		
	Soil	Water	Total
Philippines	83/106 (78%)	3/4 (75%)	86/110 (78%)
Japan	13/31 (42%)	2/5 (40%)	15/36 (42%)
			
Total	96/137 (70%)	5/9 (56%)	101/146 (69%)

To confirm the long-term survival of leptospires in the soil, 12 selected *Leptospira*-positive soil samples were stored in closed plastic conical tubes at room temperature (Table 2). After a certain period of time, the presence of leptospires was examined by the reisolation of the bacteria. Although the incubation time before reisolation varied among samples, leptospires were reisolated from all tested soil samples for up to 379 days after the incubation regardless of harboring the *fla*B gene. Therefore, soil is potentially one of the reservoirs for both pathogenic and saprophytic leptospires.

**TABLE 2:** Reisolation of *Leptospira* after long incubation^*a*^

Sample name	*fla*B PCR result	Date of initial isolation	Date of reisolation	Days of incubation
LES-2	+	28 January 2014	11 February 2015	379
LES-5	+	28 January 2014	19 July 2014	172
LES-6	+	28 January 2014	11 February 2015	379
LES-9	+	28 January 2014	11 February 2015	379
LES-12	+	29 January 2014	19 July 2014	171
LES-14	+	29 January 2014	11 February 2015	378
LES-17	+	29 January 2014	11 February 2015	378
LES-23	+	29 January 2014	11 February 2015	378
PCC I-4	−	27 May 2014	11 February 2015	260
PCC I-5	−	27 May 2014	11 February 2015	260
PCC I-13	−	27 May 2014	11 February 2015	260
PCC I-16	−	27 May 2014	10 October 2014	136

a The clade belonging to each isolate is indicated by the results of PCR for *fla*B. A positive indicates the pathogenic clade, while a negative indicates the saprophtic clade.

### Leptospires multiply in waterlogged soil.

**FIG 1:**
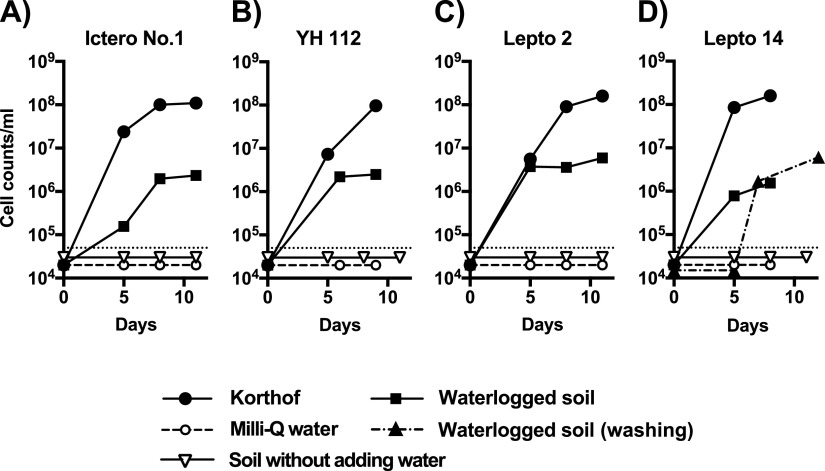
Growth of leptospires in waterlogged soil. Leptospiral strains, namely, Ictero No. 1 (A), YH 112 (B), Lepto 2 (C), and Lepto 14 (D), were subjected to growth experiments using Korthof’s medium, waterlogged soil, soil without adding water and Milli-Q water. Cell numbers of culture supernatant for up to 12 days were counted by dark-field microscopy. These experiments were repeated at least three times, and the representative data are shown in this figure. The *x* axis represents the date from the start of culture, and the *y* axis represents the cell concentration of *Leptospira* in log scale.

Since postflooding soil conditions might be suitable for growing leptospires, a growth experiment of *Leptospira* using waterlogged soils, a 1:2.5 mixture of soil and water, was conducted (Fig. 1). In this experiment, four strains were selected from different subclades (Fig. S1). In Korthof’s medium, all strains grew as expected and reached a cell count of more than 10^8^ cells/mL by day 8. In sterile Milli-Q water, the count of leptospires was below the detectable limit (60,000 cells/mL) at all the time points, indicating that leptospires do not multiply in Milli-Q water. When leptospires were inoculated into waterlogged ranch soil, the cell count of the four *Leptospira* strains used in this experiment increased to more than 10^6^ cells/mL by day 8 (Fig. 1A to D), although the growth rate was lower than that in Korthof’s medium. Comparing growth at day 5 of seeding, which is considered the logarithmic growth phase, Ictero No. 1 increased the count only to 1.56 × 10^5^ cells/mL (Fig. 1A), whereas Lepto 2 reached a cell count of 3.75 × 10^6^ cells/mL (Fig. 1C), suggesting that the growth speed in the waterlogged soil varies among strains. This experiment was repeated multiple times, and the growth of leptospires in waterlogged soil was observed consistently. However, the growth rate of leptospires varied among experiments and sometimes reached a cell count of more than 10^7^ cells/mL in waterlogged soil. We also conducted the growth experiment using autoclaved soils without adding water. Although it might be difficult to make a direct comparison with the waterlogged soil because of the completely different method of collecting the supernatant from soil, we could not detect that these *Leptospira* strains grew (less than 60,000 cells/mL) in the autoclaved soil without water, as shown in Fig. 1.

Considering the effect of carrying over Korthof’s medium contained in bacterial stock solutions on leptospiral proliferation, a growth experiment of *Leptospira* strain Lepto 14 was conducted in waterlogged soil after washing with Milli-Q water (Fig. 1D). Although the count of Lepto 14 was under the detection limit (60,000 cells/mL) on day 5 of incubation, it reached 1.7 × 10^6^ cells/mL on day 7. As for the reason for the lower initial growth when seeding the bacteria after washing, we cannot exclude the possibility that the medium in bacterial stock solutions is involved in the growth at an initial phase, but the cells may have been damaged by washing and centrifuging, possibly decreasing their viability.

**FIG 2:**
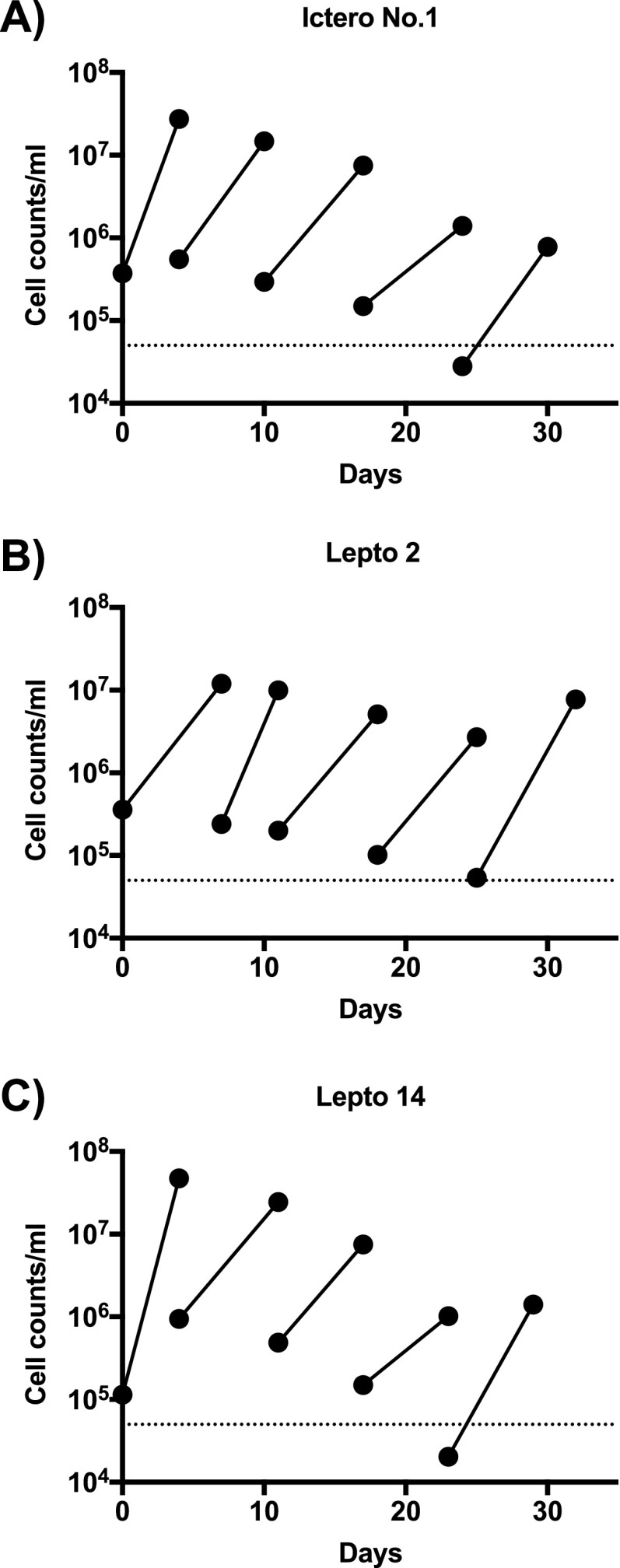
Multiple passages of leptospires in waterlogged soil. Leptospiral strains, namely, Ictero No. 1 (A), Lepto 2 (B), and Lepto 14 (C), were passed five times in waterlogged soil. Each strain was cultured in waterlogged soil for up to 9 days, and then 500 μL (1/50) of the supernatant was passed on to the next waterlogged soil sample. The *x* axis represents the date from the start of culture, and the *y* axis represents the cell concentration of *Leptospira* in log scale.

In order to examine the point without damaging the cells by washing, we conducted growth experiments in which leptospires were repeatedly passed in waterlogged soil until the remaining Korthof’s medium was fully diluted. Ictero No. 1, Lepto 2, and Lepto 14 increased their numbers even after five passages, which gave 6.25 × 10^6^-fold dilution of the original stock solution (Fig. 2), although the number of leptospiral cells in each strain tended to decrease with each passage, probably due to overdilution. The average doubling time for each strain was 29.2, 35.4, and 32.8 h, respectively, while the doubling time of leptospires in the liquid medium was between 8 and 20 h. Although the growth rate in waterlogged soil was lower than that in the growth medium, such as Korthof’s medium, our results indicated that leptospires in both pathogenic and saprophytic clades are capable of multiplying in a postflood environment like waterlogged soil.

## DISCUSSION

We have been conducting surveillance on leptospirosis in the Philippines since 1998 (5, 15, 28–36). In these surveillance studies, both pathogenic and saprophytic clades of *Leptospira* were frequently isolated from soils in the Philippines. However, no evidence of leptospiral growth in soil had been obtained from the numerous *in vitro* experiments using soils. The importance of water for the bacterial growth was suggested by previous observations: (i) leptospirosis in the Philippines occurs during the rainy season (15), (ii) occurrence of leptospirosis is higher in males working outdoors, many of whom had been exposed to flood waters (15), and (iii) the high isolation rate of leptospires from environmental samples is associated with higher content of water and fatty acids in samples (37). These facts encouraged us to conduct a series of leptospiral growth experiments in the presence of both water and soil, and we herein proved its proliferation in the waterlogged soil, a 1:2.5 mixture of soil and water, which mimics postflood conditions. Given no increase in the bacterial number in water in this study, leptospires are considered to require the soil for energy sources, such as long-chain fatty acids for optimal growth (38, 39). In a previous study, the multiplication of an Leptospira interrogans strain was not observed in water-saturated soil containing equal amounts of soil and water (12). Therefore, there seems to be an appropriate soil/water ratio for leptospiral growth. It is necessary to further examine the appropriate conditions for growth.

The present results also demonstrated that leptospires can be isolated from the soil even after an incubation for a long period of time, up to 379 days, in the absence of a sufficient amount of water to grow (soil with a typical moisture content of about 45%). In conjunction with a previous study representing that leptospires were isolated even from dry soil samples (29), we can assume that leptospires survive in a resting state in the soil without water. In contrast, leptospires may become active for multiplication in flooded conditions as described earlier. The seasonality of leptospirosis may be explained by the difference in precipitation between the rainy and dry seasons (15). To confirm this point, this work should be extended to virulent strains that have already been shown to be highly pathogenic in animals. However, since virulent strains have been reported to lose their virulence by passaging *in vitro*, such as resistance to the host’s complement system (40), growth experiments of virulent strains in the waterlogged soil are difficult at present. Since different strains may have different properties, more strains need to be investigated in the future. Our results may suggest the necessity of adaptation for *in vitro* multiplication as in L. interrogans Ictero No. 1 strain. L. interrogans Ictero No.1, which belongs to a pathogenic clade and showed multiplication in waterlogged soil in this study, had lost its pathogenicity during prolonged *in vitro* passages. There might be a tradeoff between *in vitro* and *in vivo* multiplication abilities. Regardless, the relationship between the pathogenicity of *Leptospira* and its growth in the environment could not be fully concluded in this study. The multiplication ability of virulent leptospires needs to be further investigated to understand their whole life cycle in animals and the environment.

Because leptospires survive for a prolonged period in soil and multiply in waterlogged soil, and because leptospirosis is more frequent after floods, leptospirosis may be an environment-borne disease as well as a zoonotic disease, and the ecology of the entire genus *Leptospira* should be reconsidered.

## MATERIALS AND METHODS

### Isolation of leptospires from environmental samples.

Isolation of leptospires was attempted from environmental soil and water collected in various locations in the Philippines and Japan during different seasons. The date and location information (city name and latitude/longitude) of the *Leptospira* strain isolation are shown in Table S1. Soil and water samples (approximately 10 g and 10 mL, respectively) were collected in sterile 15-mL screw-cap tubes. For soil samples, 10 mL of sterile water was added and mixed. Each tube containing 10 g of soil sample soaked in 10 mL of water or 10 mL of water sample alone was incubated in a vertical position for 1 h to allow the sediments to settle. Then, 2.0 mL of supernatant from the sample was added to 2.5 mL of 2-fold concentrated Korthof’s medium (Thomas scientific, Swedesboro, NJ, USA) supplemented with 500 μL of 10-fold concentrated antimicrobial reagent called STAFF (400 μg/mL of sulfamethoxazole, 200 μg/mL of trimethoprim, 50 μg/mL of amphotericin B, 4 mg/mL of fosfomycin, and 1 mg/mL of 5-fluorouracil) (41). All antimicrobial agents were purchased from Sigma-Aldrich (St. Louis, MO, USA). These tubes were incubated at 30°C, and droplet aliquots taken from each tube culture were observed daily by dark-field microscopy for the presence of *Leptospira*, confirmed by the characteristic thin helical structures with prominent hooked ends and motility. Samples were considered negative and discarded if no *Leptospira* was detected after 28 days of incubation. When *Leptospira* was confirmed microscopically, the whole volume of sample was filtered using a 0.2-μm-pore-size membrane filter to remove the contaminants (other microbes such as bacteria and fungi). A half milliliter of the filtrate was then added to 4.5 mL of fresh Korthof’s medium or semisolid medium containing 0.1% (wt/vol) agar without STAFF and cultured at 30°C. The number of the bacteria was counted using a Petroff-Hausser bacterial cell-counting chamber under dark-field microscopy. Single-colony isolation was performed in solid Korthof’s medium containing 1% (wt/vol) agar. Bacteria in the liquid culture medium were diluted to 10^4^ cells/mL and inoculated over the surface of the solid medium with a glass spreader. The inoculated plate was incubated at 30°C and observed daily for the appearance of subsurface colonies to pick a single colony up. The selected strains were purely cultured in Korthof’s medium to prepare bacterial stocks, and their aliquots were stored at −80°C for growth experiments.

### DNA extraction.

Bacterial DNA was extracted from 0.5 mL of the primary culture before filtration and 0.1 mL of the pure confluent culture of *Leptospira* isolates using an Illustra bacteria genomic prep mini spin kit (GE Healthcare, Chalfont St Giles, Buckinghamshire, United Kingdom) following the protocol designated for Gram-negative bacteria. Pure confluent culture of *Leptospira* strain Ictero No. 1 and Milli-Q water were used as positive and negative controls for the following PCR, respectively.

### 23S rRNA (*rrl*) gene PCR and *fla*B nested PCR.

It has been reported that all *Leptospira* species can be identified by detecting 23S rRNA (*rrl*) gene via PCR (42). For the 23S rRNA gene PCR, the primers rrl-F (5′-GACCCGAAGCCTGTCGAG-3′) and rrl-R (5′-GCCATGCTTAGTCCCGATTAC-3′) were used (28). Leptospiral DNA from the samples was amplified in a thermal cycler under the following conditions: 30 cycles of 94°C for 20 s, 54°C for 30 s, and 72°C for 1 min and an extension at 72°C for 6 min using *Ex Taq* HS DNA polymerase (TaKaRa, Otsu, Shiga, Japan). PCR products were checked by electrophoresis on 1.5% (wt/vol) agarose gel.

The *fla*B nested PCR for detecting leptospires in a pathogenic clade was performed as reported previously (7). For the first PCR, the primers l-flaB-F1 (5′-CTCACCGTTCTCTAAAGTTCAAC-3′) and l-flaB-R1 (5′-TGAATTCGGTTTCATATTTGCC-3′) were used (29). PCR amplification was performed under the following conditions: an initial cycle of 1 min at 94°C and 25 cycles at 94°C for 10 s, 50°C for 30 s, and 72°C for 1 min using *Ex Taq* HS DNA polymerase (TaKaRa). Then, 1 μL of the first-round PCR product was added to 19 μL of the second-round PCR mixture. For the second PCR, the primers l-flaB-F2 (5′-TGTGCACAAGACGATGAAAGC-3′) and l-flaB-R2 (5′-AACATTGCCGTACCACTCTG-3′) were used (29). The second PCR amplification consisted of an initial cycle of 1 min at 94°C, 30 cycles for 10 s at 94°C, 30 s at 55°C, and 50 s at 72°C and a final extension for 7 min at 72°C using *Ex Taq* HS DNA polymerase (TaKaRa). PCR products were checked to confirm the detection of *fla*B by electrophoresis on 1.5% (wt/vol) agarose gels. The PCR was conducted with reference to the “Quality Assurance/Quality Control Guidance for Laboratories Performing PCR Analyses on Environmental Samples (EPA 815-B-04-001)” issued by U.S. Environmental Protection Agency.

### 16S rRNA (*rrs*) gene sequencing and phylogenetic analysis.

The 16S rRNA gene was amplified with bacterial universal primers P16S-8UA (5′-AGAGTTTGATCMTGGCTCAG-3′) and P16S-1485R (5′-TACGGYTACCTTGTTACGACTT-3′) using *Ex Taq* HS DNA polymerase (TaKaRa) (29). PCR amplification was performed under the following conditions: 30 cycles at 96°C for 1 min, 55°C for 1 min, and 72°C for 1.5 min. After the amplicons of the 16S rRNA gene by electrophoresis on 1% (wt/vol) agarose gels were confirmed, the PCR products were purified by using a PCR purification kit (Qiagen, Venlo, Netherlands), and the sequence was determined using a 3130 genetic analyzer (Thermo Fisher Scientific, Waltham, MA, USA). The 16S sequences of *Leptospira* obtained in this study were deposited in GenBank (accession numbers MW727617, MW727618, MW727619, and MW727620). The sequence data were compared with those of representative reference strains collected from public databases (GenBank, NCBI, NIH) and phylogenetically analyzed using GENETYX Network v.13.1.1 (GENETYX). According to the Bayesian Information Criterion (43), the model with the lowest score was used in maximum likelihood analysis to establish the best-fit substitution model. Phylogenetic trees were constructed using MEGA v.7.0 (http://www.megasoftware.net/) under the best-fit model.

### Survival test of *Leptospira* in soil.

The long-term survival test was conducted with 100 g of 12 selected soil samples positive for leptospires. Initially, leptospires were isolated from 10 g of the soil and cultured in Korthof’s medium as described above, followed by the identification of *rrl* and *flaB* genes by PCR. Then, the remaining 90 g of the soil samples with confirmed *Leptospira* isolation was stored in a closed conical tube at room temperature (24 to 26°C) for the indicated period. Subsequently, isolation and identification of *Leptospira* were attempted from 10 g of soil in the remaining samples as described above.

### Growth experiments in waterlogged soil.

To examine whether leptospires multiply in the environmental samples without the animal host, *in vitro* growth experiments were conducted in water, soil, and waterlogged soil (mimicking the situation after floods). We used soil from a ranch in Hokkaido University Campus after it was autoclaved to sterilize any potential leptospires and other bacteria in the soil, as well as sterile Milli-Q water. The moisture content of the ranch soil was 45% (mean value from two experiments), which was calculated from the weight before and after drying as reported previously (29), and the pH examined by paper pH strips (Thermo Fisher Scientific) was 6.8 (mean value from three experiments; range was 6.5 to 7.0).

For this experiment, the following four strains were selected from the P1, P2, S1, and S2 subclades, respectively: L. interrogans serovar Icterohaemorrhagiae strain Ictero No. 1 kindly provided by Y. Kobayashi, L. wolffii strain YH 112 (sample number 115), L. yanagawae strain Lepto 2 (sample number 130), and Lepto 14 (sample number 122) closely related to L. ryugenii (Table S1 and Fig. S1). The *Leptospira* stock culture was diluted in Milli-Q water to make the concentration to 1 × 10^6^ cells/mL.

In growth experiments, 5 × 10^5^ cells in 500 μL of the diluted stock were inoculated into 100-mL glass bottles containing 25 mL of sterile Milli-Q water (water bottle) or 10 g of autoclaved soil without or with 25 mL of sterile Milli-Q water (soil bottle or waterlogged soil bottle, respectively). In addition, 25 mL of Korthof’s medium without soil was prepared in the bottle as positive control. The closed bottles were incubated at room temperature (24 to 26°C) for up to 12 days. A small portion (about 100 μL) of the supernatant was aseptically collected continuously from each bottle at different time points. To collect leptospires from the soil bottle at each indicated time point, soil was resuspended in 25 mL of water for 30 min with gentle agitation, and then 100 μL of the supernatant was collected. The number of leptospires in collected supernatants was counted using a Petroff-Hausser bacterial cell-counting chamber under dark-field microscopy. The detection limit of cells was 60,000 cells/mL. The reproducibility was confirmed by at least three repeated experiments.

To exclude the possibility that bacterial growth was supported by culture medium from the stock, *Leptospira* stock culture of Lepto 14 was centrifuged (4,400 × *g*, 20 min) and washed twice with Milli-Q water to remove the remaining Korthof’s medium. The washed cells were diluted to 6 log cell counts/mL with Milli-Q water, 500 μL (5 × 10^5^ cells) was inoculated into waterlogged soil, and the cell count was performed as described above.

In addition, transplanting tests were conducted to reduce the effect of Korthof’s medium in the initial inoculation by serial dilution on the leptospiral growth in waterlogged soil. After inoculation of Ictero No.1, Lepto 2, and Lepto 14 in waterlogged soil bottles, 500 μL of the supernatant was transferred into a new waterlogged soil bottle every 4 to 6 days, which means 50-fold dilution per passage. The passage was repeated four times, and finally the medium contained in the initial inoculum was diluted by 6.25 × 10^6^-fold. Six days after the last passage, the cell number in the supernatant was measured as described above.

### Data availability.

The 16S sequences of *Leptospira* obtained in this study were deposited in GenBank (accession numbers MW727617, MW727618, MW727619, and MW727620).
