# Supplementary material for: Leptospira Is an Environmental Bacterium That Grows in Waterlogged Soil
Source: Microbiol Spectr. 2022 Mar 15;10(2):e02157-21. doi: 10.1128/spectrum.02157-21 (PMC9045322; doi:10.1128/spectrum.02157-21)
Supplement: SUPPLEMENTAL FILE 1 — Supplemental material. Download SPECTRUM02157-21_Supp_1_seq8.pdf, PDF file, 2.2 MB [file spectrum02157-21_supp_1_seq8.pdf]

Detection of leptospires from the environment in Philippines

Sample No.	Sampling Date	Sample Name	Source	Location (City)	Latitude and longitude	Elevation	rrl PCR	Isolation	*fla*B PCR
1	January 28, 2014	LES-1	Soil	Tacloban	11°13'10"N, 125°01' 09"E	0 m	+	+	-
2		LES-2	Soil		11°13'07"N, 125°00' 12"E	0 m	+	+	+
3		LES-3	Soil		11°12'57"N, 125°01' 21"E	0 m	+	+	-
4		LES-4	Soil		11°12'45"N, 125°01' 26"E	0 m	-	-	n.t.
5		LES-5	Soil		11°15'03"N, 125°00' 08"E	2 m	+	+	+
6		LES-6	Soil		11°14'43"N, 124°59' 53"E	1 m	+	+	+
7		LES-7	Soil		11°14'50"N, 124°59' 38"E	24 m	+	+	-
8		LES-8	Soil		11°14'55"N, 124°59' 33"E	25 m	+	+	-
9		LES-9	Soil		11°15'09"N, 124°59' 25"E	3 m	+	+	+
10		LES-10	Soil		11°13'27"N, 125°00' 11"E	1 m	+	+	-
11	January 29, 2014	LES-11	Soil	Bungto han Palo	11°10'21"N, 125°00' 44"E	0 m	+	+	-
12		LES-12	Soil		11°10'05"N, 125°00' 40"E	1 m	+	+	+
13		LES-13	Soil		11°10'01"N, 125°00' 25"E	1 m	+	+	+
14		LES-14	Soil		11°10'15"N, 125°00' 31"E	2 m	+	+	+
15		LES-15	Soil		11°09'12"N, 125°00' 23"E	3m	+	+	-
16		LES-16	Soil		11°09'06"N, 125°00' 34"E	2 m	+	+	-
17		LES-17	Soil		11°09'00"N, 125°00' 41"E	1 m	+	+	+
18		LES-18	Soil		11°09'30"N, 125°00' 02"E	5 m	+	+	-
19		LES-19	Soil		11°09'51"N, 125°00' 05"E	3 m	+	+	+
20		LES-20	Soil		11°09'12"N, 124°59' 46"E	9 m	+	+	-
21		LES-21	Soil		11°09'12"N, 124°59' 26"E	7 m	+	+	+
22		LES-22	Soil		11°10'29"N, 125°00' 56"E	0 m	+	+	-
23		LES-23	Soil		11°10'30"N, 125°00' 50"E	0 m	+	+	+
24	February 20, 2014	Angeles Market	Soil	Angeles	n.i.	n.i.	+	+	+
25	May 27, 2014	PCC I-1.	Soil	Science City of Munoz	15°43'07"N, 120°52' 25"E	57 m	-	-	n.t.
26		PCC I-2	Soil		15°43'07"N, 120°52' 25"E	57 m	-	-	n.t.
27		PCC I-3	Soil		15°43'07"N, 120°52' 25"E	57 m	-	-	n.t.
28		PCC I-4	Soil		15°43'07"N, 120°52' 25"E	57 m	+	+	-
29		PCC I-5	Soil		15°43'07"N, 120°52' 25"E	57 m	+	+	-
30		PCC I-6	Soil		15°43'07"N, 120°52' 25"E	57 m	-	-	n.t.
31		PCC I-7	Soil		15°43'07"N, 120°52' 25"E	57 m	-	-	n.t.
32		PCC I-8	Soil		15°43'07"N, 120°52' 25"E	57 m	-	-	n.t.
33		PCC I-9	Soil		15°43'07"N, 120°52' 25"E	57 m	-	-	n.t.
34		PCC I-10	Soil		15°43'07"N, 120°52' 25"E	57 m	-	-	n.t.
35		PCC I-11	Soil		15°43'07"N, 120°52' 25"E	57 m	-	-	n.t.
36		PCC I-12	Soil		15°43'07"N, 120°52' 25"E	57 m	-	-	n.t.
37		PCC I-13	Soil		15°43'07"N, 120°52' 25"E	57 m	+	+	-
38		PCC I-14	Soil		15°43'07"N, 120°52' 25"E	57 m	-	-	n.t.
39		PCC I-15	Soil		15°43'07"N, 120°52' 25"E	57 m	-	-	n.t.
40		PCC I-16	Soil		15°43'07"N, 120°52' 25"E	57 m	+	+	-
41		PCC Vermiculture	Soil		15°43'07"N, 120°52' 25"E	57 m	+	+	-
42	June 18, 2014	Gardening Soil	Soil	Manila	n.i.	n.i.	+	+	-
43	June 19, 2014	Aloe Pot	Soil	Marikina	n.i.	n.i.	+	+	-
44	June 25, 2014	Pasig I-1	Soil	Pasig	n.i.	n.i.	+	+	-
45		Pasig I-2	Soil		n.i.	n.i.	+	+	+
46	June 25, 2014	San Fernando	Soil	Pampanga	n.i.	n.i.	+	+	-
47	June 29, 2014	Sharon 1	Soil	Manila	n.i.	n.i.	-	-	n.t.
48		Sharon 2, Bougainvillea Pot	Soil		n.i.	n.i.	+	+	-
49		Sharon 3, Bougainvillea Pot	Soil		n.i.	n.i.	+	+	+
50		Sharon 4	Soil		n.i.	n.i.	-	-	n.t.
51		Sharon 5	Soil		n.i.	n.i.	-	-	n.t.
52		Sharon 6, Magnolia Pot	Soil		n.i.	n.i.	+	+	+
53		Sharon 7, Magnolia Pot	Soil		n.i.	n.i.	+	+	-
54	June 29, 2014	Tropicana Pool side Pot 1	Soil	Manila	14°34'28"N, 120°59' 11"E	4 m	+	+	-
55		Tropicana Pool side Pot 2	Soil		14°34'28"N, 120°59' 11"E	4 m	+	+	-
56		Tropicana Pool side Compost	Soil		14°34'28"N, 120°59' 11"E	4 m	+	+	-
57		Tropicana 9FL Roof Calamansi Pot	Soil		14°34'28"N, 120°59' 11"E	4 m	+	+	+
58		Tropicana 9FL Roof Banana Pot	Soil		14°34'28"N, 120°59' 11"E	4 m	-	-	n.t.
59	July 06, 2014	Taytay 1	Soil	Taytay	n.i.	n.i.	+	+	-
60		Taytay 2	Soil		n.i.	n.i.	+	+	-
61	July 06, 2014	Pasig II-1	Soil	Pasig	n.i.	n.i.	+	+	-
62		Pasig II-2	Soil		n.i.	n.i.	+	+	-
63		Pasig II-3	Soil		n.i.	n.i.	+	+	-
64	July 07, 2014	Tropicana 8FL Hibiscus Pot	Soil	Manila	14°34'28"N, 120°59' 11"E	4 m	+	-	n.t.
65		Tropicana 8FL Flower Pot	Soil		14°34'28"N, 120°59' 11"E	4 m	+	-	n.t.
66	July 07, 2014	CPH Lara Hall	Soil	Manila	14°34'32"N, 120°59' 10"E	5 m	+	+	-
67		CPH Annex Building	Soil		14°34'35"N, 120°59' 12"E	5 m	+	+	-
68	July 07, 2014	Santa Ana 1	Soil	Manila	14°34'20"N, 120°59' 54"E	4 m	+	+	-
69		Santa Ana 2	Soil		14°34'20"N, 120°59' 54"E	4 m	+	+	-
70		Santa Ana 3	Soil		14°34'20"N, 120°59' 54"E	4 m	+	+	-
71	July 08, 2014	Makati 1	Soil	Manila	n.i.	n.i.	+	+	+
72		Makati 2	Soil		n.i.	n.i.	+	+	-
73		Makati 3	Soil		n.i.	n.i.	+	-	n.t.
74	July 16, 2014	CM UPM	Soil	Manila	14°34'32"N, 120°59' 09"E	5 m	+	+	-
75		CM UPM Surface Water 1	Water		14°34'32"N, 120°59' 09"E	5 m	+	+	-
76		CM UPM Surface Water 2	Water		14°34'32"N, 120°59' 09"E	5 m	+	+	-

Supplemental Table 1

77	July 21, 2014	UN Ave., Plant Box 1	Soil	Manila	14°34'03"N, 120°59' 06"E	3 m	+	+	-
78		UN Ave., Plant Box 2	Soil		14°34'03"N, 120°59' 06"E	3 m	+	+	-
79	July 21, 2014	Plant Box Bel	Soil	Manila	n.i.	n.i.	+	+	-
80	July 25, 2014	Fortune tree pot, Bel	Soil	Manila	n.i.	n.i.	+	+	-
81	July 27, 2014	Jonathan Cavite 1	Soil	Lungsod ng Cavite	14°28'34"N, 120°54' 03"E	0 m	+	+	-
82		Jonathan Cavite 2	Soil		14°28'34"N, 120°54' 03"E	0 m	+	+	+
83		Jonathan Cavite 3	Soil		14°28'34"N, 120°54' 03"E	0 m	+	+	-
84	September 19, 2014	K's Garden Inside 1 a	Soil	Makati	14°34'03"N, 121°01' 32"E	5 m	+	+	-
85		K's Garden Inside 1 b	Soil		14°34'03"N, 121°01' 32"E	5 m	+	+	-
86		K's Garden Outside 1 a	Soil		14°34'03"N, 121°01' 32"E	5 m	+	+	-
87		K's Garden Outside 1 b	Soil		14°34'03"N, 121°01' 32"E	5 m	+	+	-
88	September 20, 2014	Front, IMF 1a	Soil	Iloilo	10°38'27"N, 122°13' 26"E	27 m	+	+	-
89		Front, IMF 1b	Soil		10°38'27"N, 122°13' 26"E	27 m	+	+	-
90		Close to IMF sea a	Soil		10°38'27"N, 122°13' 26"E	27 m	+	+	-
91		Close to IMF sea b	Soil		10°38'27"N, 122°13' 26"E	27 m	+	+	-
92		Inside IMF 3 a	Soil		10°38'27"N, 122°13' 26"E	27 m	+	+	-
93		Inside IMF 3 b	Soil		10°38'27"N, 122°13' 26"E	27 m	+	+	-
94	October 03, 2014	DTRI 1	Soil	Los Banos	14°09'05"N, 121°14'40"E	69 m	+	+	n.t.
95		DTRI 2a	Water		14°09'05"N, 121°14'40"E	69 m	+	+	n.t.
96		DTRI 2b	Soil		14°09'05"N, 121°14'40"E	69 m	+	+	n.t.
97		DTRI 3	Soil		14°09'05"N, 121°14'40"E	69 m	+	+	n.t.
98	October 03, 2014	Tabon 1	Soil	Los Banos	14°09'46"N, 121°14'41"E	33 m	+	+	n.t.
99		Tabon 2	Soil		14°09'46"N, 121°14'41"E	33 m	+	+	n.t.
100		Tabon 3	Soil		14°09'46"N, 121°14'41"E	33 m	+	+	n.t.
101	October 03, 2014	Raymundo 1	Soil	Los Banos	14°09'05"N, 121°14'40"E	69 m	+	+	n.t.
102		Raymundo 2	Soil		14°09'05"N, 121°14'40"E	69 m	-	-	n.t.
103		Raymundo 3	Soil		14°09'05"N, 121°14'40"E	69 m	+	+	n.t.
104	October 03, 2014	Bangkai 1	Soil	San Pablo City	14°03'60"N, 121°19'23"E	109 m	-	-	n.t.
105		Bangkai 2a	Soil		14°03'60"N, 121°19'23"E	109 m	-	-	n.t.
106		Bangkai 2b	Water		14°03'60"N, 121°19'23"E	109 m	-	-	n.t.
107		Bangkai 3	Soil		14°03'60"N, 121°19'23"E	109 m	+	+	n.t.
108	October 03, 2014	Batong Malake 1	Soil	Los Banos	14°09'05"N, 121°14'40"E	69 m	+	+	n.t.
109		Batong Malake 2	Soil		14°09'05"N, 121°14'40"E	69 m	+	+	n.t.
110		Batong Malake 3 a	Soil		14°09'05"N, 121°14'40"E	69 m	+	+	n.t.

n.i.; no information   n.t.; not tested

Detection of leptospires from the environment in Japan

Sample No.	Sampling Date	Sample Name	Source	Location (City)	Latitude and longitude	Elevation	*rrl* PCR or rrs Seq	Isolation	*fla*B PCR	Name of Isolated Strain
111	August 18, 2014	Syoukouji Grass Field	Soil	Chino	36°03'11"N, 138°15'14"E	1,216 m	+	+	+	*L. alstonii* YH107
112		Syoukouji Garden 2	Soil		36°03'11"N, 138°15'14"E	1,216 m	-	-	n.t.	
113	August 20, 2014	Hokuto City, Ooizumi Cabin	Soil	Hokuto	35°54'03"N, 138°24'18"E	1,206 m	-	-	n.t.	
114		F's Kitchen Garden 1	Soil		35°54'52"N, 138°24'18"E	1,201 m	+	+	+	*L. alstonii* YH117
115		F's Kitchen Garden 2	Soil		35°54'52"N, 138°24'18"E	1,201 m	+	+	+	*L. wolffii* YH112
116	August 27, 2014	Univ. Shizuoka Herb Garden A soil	Soil	Shizuoka	34°59'35"N, 138°26'54"E	107 m	-	-	n.t.	
117		Univ. Shizuoka Herb Garden B soil	Soil		34°59'35"N, 138°26'54"E	107 m	-	-	n.t.	
118		Univ. Shizuoka Herb Garden Water	Water		34°59'35"N, 138°26'54"E	107 m	+	+	n.t.	*L. ryugenii* YH101
119	August 29, 2014	YY Home garden	Soil	Shizuoka	35°02'02"N, 138°25'30"E	51 m	-	-	n.t.	
120	October 22, 2018	Mt. Yatsugatake forest 1	Soil	Hokuto	35°54'03"N, 138°24'18"E	1,214 m	n.t.	-	n.t.	
121		Mt. Yatsugatake forest Moulding Mound	Soil		35°54'03"N, 138°24'18"E	1,214 m	n.t.	-	n.t.	
122	December 20, 2018	Humus Shizuoka 1	Soil	Shizuoka	35°02'02"N, 138°25'30"E	51 m	+	+	-	Lepto 14
123	December 23, 2018	Mt. Yatsugatake	Soil	Hokuto	35°54'03"N, 138°24'18"E	1,214 m	+	+	-	
124	January 10, 2019	Nagao River	Water	Shizuoka	35°01'52"N, 138°25'21"E	37 m	n.t.	-	n.t.	
125		Nagao River, Sandbank	Soil		35°01'52"N, 138°25'21"E	37 m	n.t.	-	n.t.	
126		Y.Y.Chrysanthemum Plqnter	Soil		35°02'02"N, 138°25'30"E	51 m	n.t.	-	n.t.	
127		Y.Y.Chionanthus Retusus Planter	Soil		35°02'02"N, 138°25'30"E	51 m	n.t.	-	n.t.	
128	January 23, 2019	Humus, Sapporo	Soil	Sapporo	43°04'50"N, 141°20'10"E	10 m	n.t.	-	n.t.	
129	January 24, 2019	Hokudai Horse Ranch	Soil	Sapporo	43°04'52"N, 141°20'11"E	10 m	+	+	-	
130		Hokudai Catttle Ranch	Soil		43°04'52"N, 141°20'11"E	10 m	+	+	-	Lepto 2
131		Hokudai Compost	Soil		43°04'53"N, 141°20'11"E	10 m	+	+	-	
132		Hokudai Recreation Field	Soil		43°04'52"N, 141°20'11"E	10 m	n.t.	-	n.t.	
133	February 24, 2019	Humus Shizuoka 2	Soil	Shizuoka	35°02'02"N, 138°25'30"E	51 m	n.t.	-	n.t.	
134	February 25, 2019	Mt. Yatsugatake forest 2	Soil	Hokuto	35°54'03"N, 138°24'18"E	1,214 m	+	+	-	
136	April 03, 2019	Hokudai Cattle Ranch 1	Soil	Sapporo	43°04'52"N, 141°20'11"E	10 m	+	+	-	
137		Hokudai Cattle Ranch 2	Soil		43°04'52"N, 141°20'11"E	10 m	+	+	-	
138		Hokudai Recreation Field	Soil		43°04'51"N, 141°20'12"E	10 m	n.t.	-	n.t.	
139		Hokudai 2nd Agr Field Pond	Water		43°04'53"N, 141°20'26"E	10 m	n.t.	-	n.t.	
140		Hokudai 2nd Agr Field	Soil		43°04'53"N, 141°20'26"E	10 m	n.t.	-	n.t.	
141		Hokudai Onoike Pond	Water		43°04'27"N, 141°20'30"E	10 m	+	+	-	
142		Hokudai Sakushukotoni River	Water		43°04'27"N, 141°20'30"E	10 m	n.t.	-	n.t.	
143		Hokudai Sakushukotoni Riverside	Soil		43°04'27"N, 141°20'30"E	10 m	n.t.	-	n.t.	
144		Hokudai Archery Hall	Soil		43°04'26"N, 141°20'36"E	11 m	+	+	-	
145		Hokudai CZC Field 1	Soil		43°04'57"N, 141°20'10"E	10 m	+	+	-	
146		Hokudai CZC Field 2	Soil		43°05'00"N, 141°20'12"E	10 m	n.t.	-	n.t.	

n.t.; not tested

Supplemental Table 1 - continued

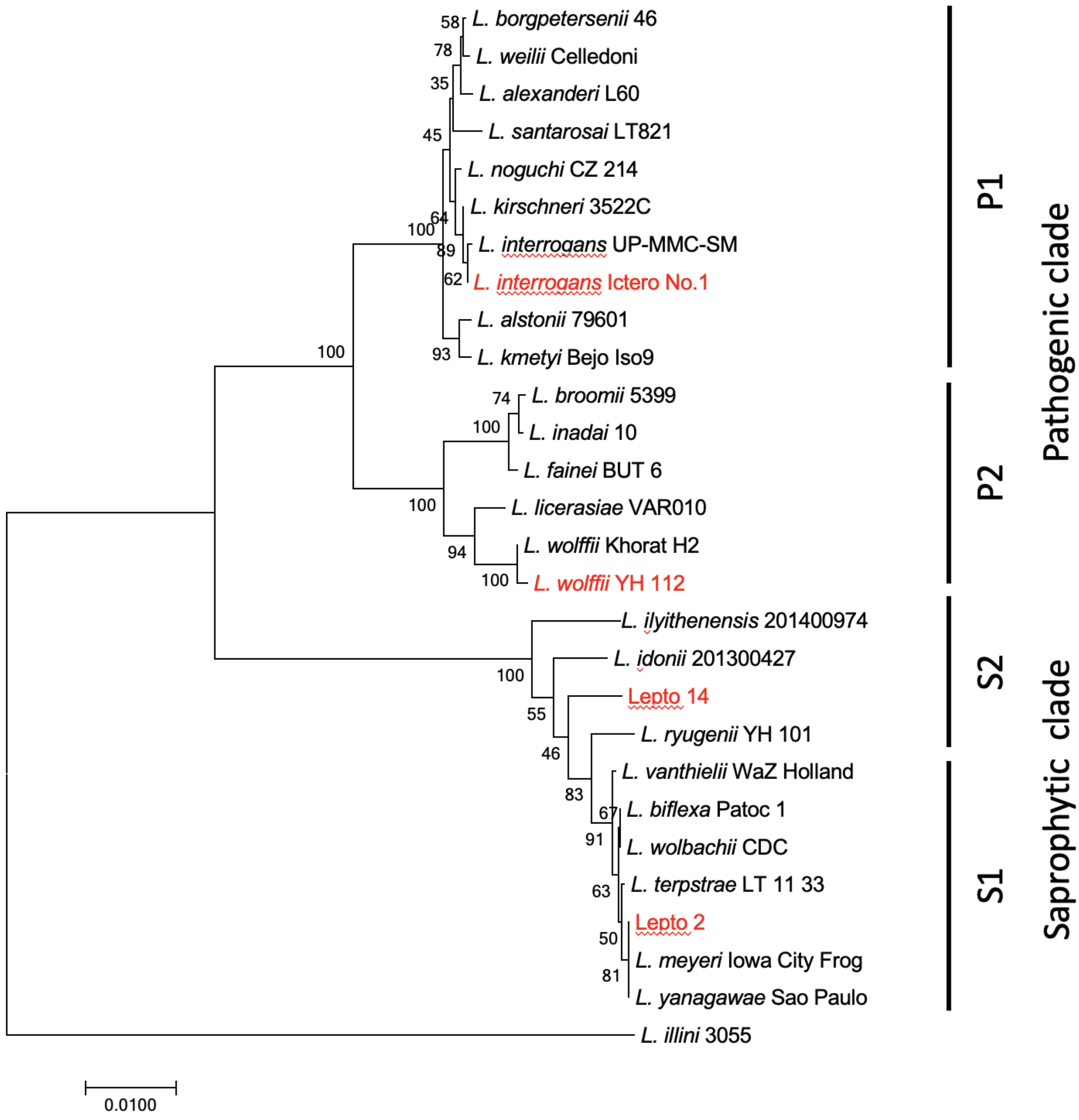

Supplemental Figure 1
Subclades determined by phylogenetic analysis of Leptospira strains used in this study.

The phylogenetic tree based on partial 16S ribosomal RNA gene sequences of leptospires was constructed by the maximum likelihood method using MEGA v.7.0 under the best-fit model. Clades and subclades are shown on the right. The strains used in this study are highlighted in red.

Supplemental Figure 1
